# Paraphilic Disorder in a Male Patient with Autism Spectrum Disorder: Incidence or Coincidence

**DOI:** 10.7759/cureus.2639

**Published:** 2018-05-16

**Authors:** Bishoy Kolta, Garrett Rossi

**Affiliations:** 1 Psychiatry, Copper University Hospital, Camden, USA; 2 Psychiatry, Cooper University Hospital, Camden, USA

**Keywords:** autism spectrum disorders, paraphilias, child psychiatry, developmental psychiatry, psychiatry

## Abstract

Paraphilic disorder in patients with autism spectrum disorder (ASD) can be extremely disturbing to both the patient and caretakers. It can interfere with these patient’s ability to develop social skills, which are essential for adaptation, and function within society. This case report details the history of an 18-year-old male patient previously diagnosed with Asperger’s syndrome, who also exhibited symptoms consistent with paraphilic disorder. A review of the literature was then conducted to determine if there is any documented association of paraphilic disorder, or abnormal sexual behavior in patients with ASD. Several case reports involving patients with ASD, and co-morbid paraphilic disorder have been described in the literature. ASD appears to be associated with paraphilic disorder.

## Introduction

The Diagnostic and Statistical Manual fifth edition (DSM-5) describes paraphilia as recurrent, and intense sexually arousing fantasies, sexual urges, or behavior that occurs over a period of six months causing clinically significant distress, impairment in social, occupational, or other important areas of function [1]. Autism spectrum disorder (ASD) is a neurodevelopmental disorder characterized by marked impairment in language, and social interaction with repetitive stereotyped behavior [1]. Very few studies have been published on ASD and its relationship to paraphilia, and majority of the published information comes from case histories. Studies that have been carried out on sexuality and ASD demonstrate that patients with ASD have a wide variety of sexual behaviors [2-4]. There has been an increase in the reported frequency of ASD in the United States with a frequency approaching 1% of the population [1]. The prevalence of paraphilias is not well established in the literature. The reason for this is likely related to reluctance on the part of the patient to disclose these behaviors as they are embarrassing, and in some cases against the law. Clinicians may not ask about sexual behaviors of their patients if the initial presentation does not lead to that line of questioning. As per the DSM-5, the frequency of voyeuristic disorder is unknown, but the estimated lifetime prevalence is approximately 12% for males and 4% for females [1]. The frequency of exhibitionistic disorder is also unknown, but the prevalence in males is estimated to be 2-4% [1]. Frotteuristic disorder may occur in as many as 30% of adult males in the general population [1]. The frequency of sexual masochism disorder is unknown. We report the case of an 18-year-old male patient diagnosed with ASD, who also exhibited paraphilic behavior and fantasies. Based on a thorough search of the literature, very few cases of patients with ASD, and comorbid paraphilic disorder have been described. We selected 11 publications for review with the goal of demonstrating a correlation between ASD and paraphilia.

## Case presentation

This case details the history of an 18-year-old Caucasian male, with a past psychiatric history of ASD, who initially presented to the psychiatric emergency service with complaints of depressed mood, and suicidal ideation with a plan to hang himself. The patient reported that he put a rope around his neck, and was about to kill himself, however, he had second thoughts, and walked into the hospital asking for help. The patient reported having these thoughts after experiencing sexual fantasies. These fantasies included being aroused by "anthropomorphic animal characters" and were self-described as "furry". He had a self-reported history of having a violent sexual fantasy in which he "had sex with a girl and then cut off her head." The patient reported two previous suicidal attempts, the first being when he was 16 years of age. The patient described trying to strangle himself with his hands, but denied seeking any medical attention. The patient's second and most severe suicide attempt occurred a few weeks prior to his presentation at the psychiatric emergency service, after having a violent sexual fantasy in which he "had sex with a girl and cut off her head." The patient was deeply disturbed by this fantasy, and he experienced intense fear, anxiety and guilt as a result. These intense feelings led to his suicide attempt in which he tried to suffocate himself with a plastic bag.

On psychiatric review of symptoms, the patient endorsed the following neurovegetative symptoms of depression including poor sleep, a recent loss of interest, difficulty concentrating, guilt over a recent sexual fantasy and his perceived inability to socialize like his peers. He denied loss of energy, change in appetite, psychomotor retardation and feeling hopeless, or helpless.

**Figure 1:**
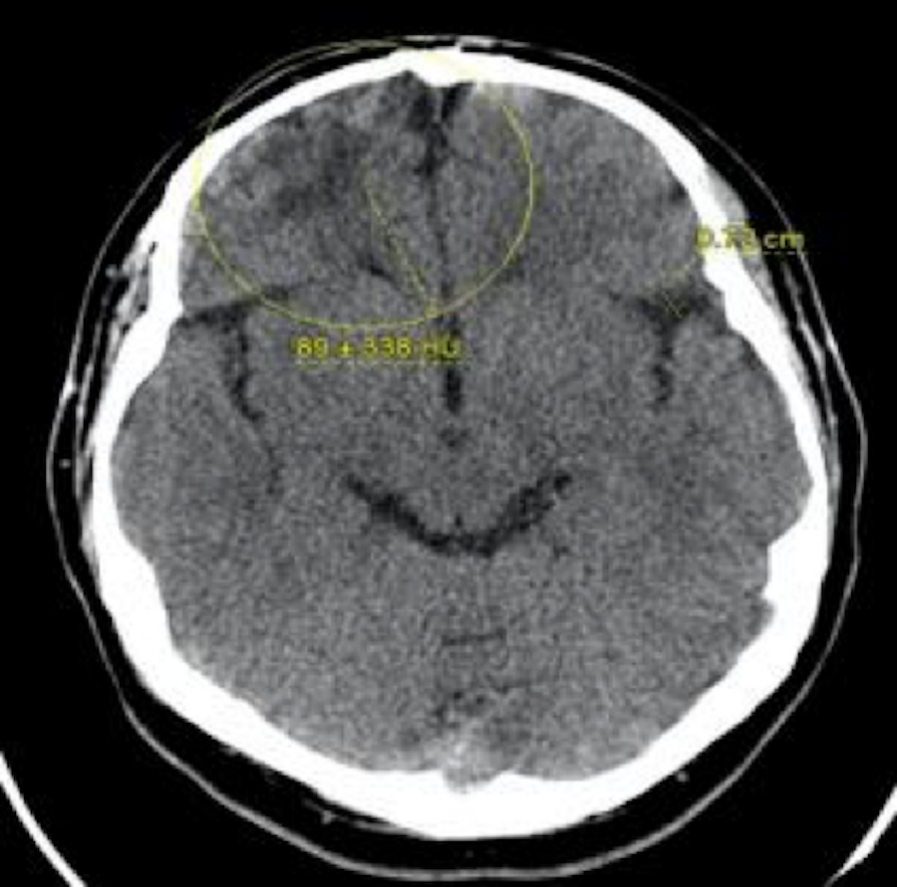
Computed tomography head without contrast coronal view. Yellow circle indicates area of encephalomalacia in right frontal lobe.

**Figure 2:**
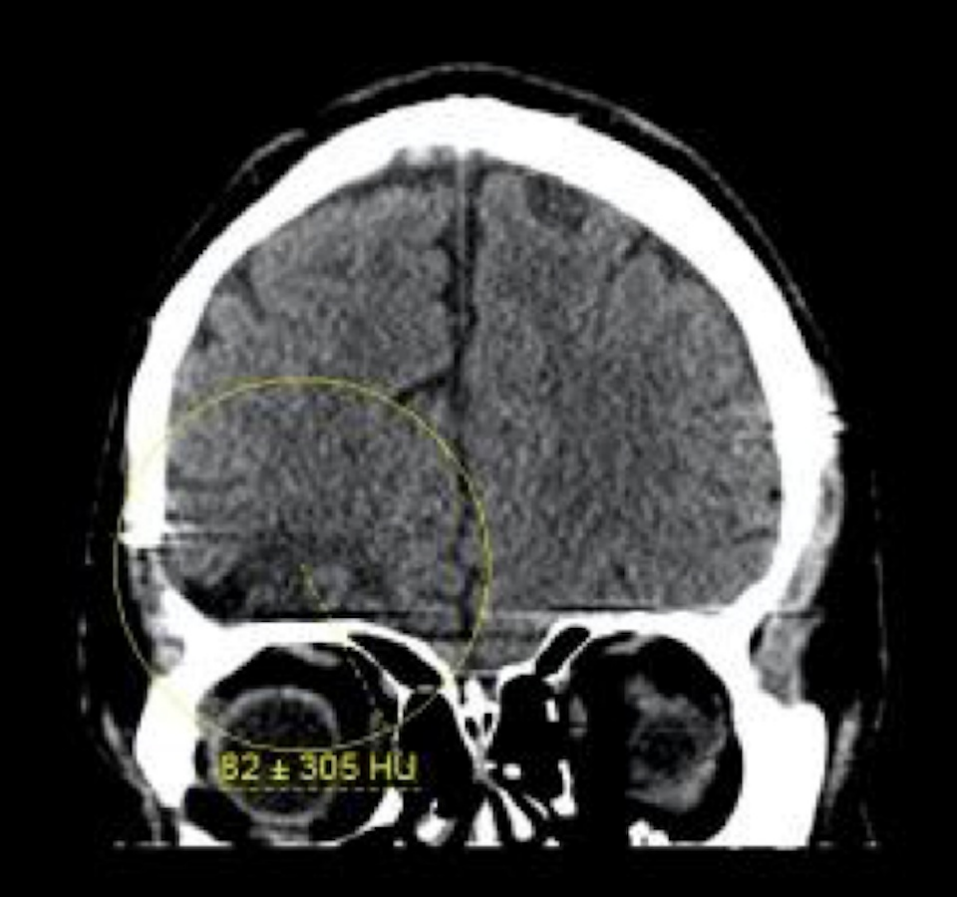
Computed tomography head without contrast axial view. Yellow circle indicates area of encephalomalacia in right temporal lobe.

The patient had a significant past medical history for sinusitis at the age of ten complicated by a brain abscess that required a computed tomography (CT) guided craniotomy for drainage of the abscess. The patient had a repeat CT scan without contrast that showed encephalomalacia on the axial section located in the right frontal lobe as indicated by the yellow circle in Figure 1. Figure 2 is a corronal CT scan without contrast that showed encephalomalacia located in the right temporal lobe.

## Discussion

Fernandes et al. were able to demonstrate that 30% of the low-functioning ASD patients showed some kind of inappropriate sexual behaviors, most frequently public masturbation, indecent exposure, and inappropriate heterosexual behaviors [5]. When looking at higher functioning patients with autism spectrum disorder they reported 10% demonstrated inappropriate sexual behaviors [5]. The study also revealed that 24% of high-functioning individuals with ASD engaged in paraphilic sexual fantasies or behaviors including classic presentations of paraphilic disorder such as pedophilia, voyeurism, and sadomasochism [5].

There are some studies exploring gender-specific aspects of hypersexual and paraphilic behaviors in patients with ASD. One study looked at these behaviors in a cohort of patients with high functioning ASD, and concluded that these patients are more hypersexual and have more paraphilic fantasies than heterosexual controls [6]. They also found that hypersexual behaviors were more common in patients with ASD, but the finding was mainly driven by the male patients with ASD, and no difference was found in the female groups [6]. Another study looked at a group of caregivers of 24 institutionalized males, high-functioning adolescents and young adults with ASD, who were interviewed using the Interview Sexuality Autism [7]. This study demonstrated the presence of paraphilia in two out of the 24 subjects. The two subjects were primarily attracted to young prepubescent girls. One had a platonic interest in young girls, while the other met the criteria for pedophilia. The other subject with a specific interest in a particular object met the criteria for fetishism.

Silva et al. made an association between ASD and sexual serial homicidal behavior [8]. Many sexual serial killers have a high prevalence of deconstructive paraphilia. They hypothesized that among autistic serial killers, this deconstructive paraphilic pattern may be a partial, but intrinsic outcome of the tendency of the autistic person to focus on objects with a relative disregard for person’s mental qualities [8].

Individuals who suffer from ASD tend to suffer paraphilic psychopathologies. The etiology of autism and paraphilia disorder is still unclear. Neurobiological approaches emphasize brain networks being disturbed in both disorders [9]. Pathologies of the amygdala and hippocampus are thought to be involved in both autism and fetishism, although no definitive studies have been conducted.

Few studies to date have made treatment recommendations for patients with ASD, and comorbid paraphilia. Kafka and Prentky showed that patients who exhibited paraphilic, and non-paraphilic sexual addiction with co-morbid mood disorders such as depression benefited from treatment with fluoxetine. Kafka and Prentky hypothesized that increased central serotonin may be responsible for the alleviation of symptoms in these patients [10]. Coskun et al. presented a case series designed to look at the effectiveness of mirtazapine in the treatment of abnormal sexual behavior (ASB) in patients with ASD. The study looked at 10 individuals (two females and eight males) averaging between 5.2 and 16.4 years of age with a primary diagnosis of ASD and who developed ASB. Mirtazapine was started at the dosage of 7.5-15 mg/day, and titrated up to 30 mg/day [11]. The symptoms and efficacy of the treatment were assessed using Clinical Global Impressions-Severity (CGI-S), which was administered at the beginning and end of treatment. Majority of the patients showed improvement in ASB. Five subjects showed considerable improvement, three subjects showed improvement and one subject showed some improvement in excessive masturbation. Six subjects showed improvement in other ASBs such as fetishism [11]. Mirtazapine seems to be effective at treating ASB and may be a favorable first-line therapy for patients with ASD and ASB after further investigations confirm these findings.

## Conclusions

The presence of paraphilic disorder in patients diagnosed with ASD is not typical considering the high frequency of ASD in the population. There is a wide variety of sexual behavior recorded in the literature in this patient population, but not all are pathological. This patient did exhibit the typical characteristics of paraphilic disorder which usually includes an atypical focus of sexual arousal that is recurrent, intense, and occurs for at least six months. It is possible that the frontal lobe damage sustained in this patient contributed to these behaviors, and should be taken into account. There are still many questions to be answered with respect to the normalcy of such behavior, and whether it is also present in female patients with ASD. The most difficult task for a clinician is figuring out the appropriate management of this condition. There is limited information regarding pharmacological, or behavioral treatments for this unique situation. A review of the literature revealed the possibility of using behavioral modification techniques in conjunction with education about healthy sexual behavior as treatment. Patients with significant distress and loss of function may warrant a more rapid form of relief. A selective serotonin reuptake inhibitor (SSRI) could help with the symptoms of depression, and have been shown to be effective in reducing paraphilic and non-paraphilic sexual addiction in men with co-morbid mood disorders. A more recent study elected to use mirtazapine in the treatment of a patient with ASD and fetishism. Mirtazapine has several benefits including anti-libidinal effects, as well as benefits in treating aggression and sleep disturbances in patients with ASD.
